# Characterization of *lpaH2* gene corresponding to lipopeptide synthesis in *Bacillus amyloliquefaciens* HAB-2

**DOI:** 10.1186/s12866-017-1134-z

**Published:** 2017-12-04

**Authors:** Pengfei Jin, Haonan Wang, Wenbo Liu, Weiguo Miao

**Affiliations:** Institute of Tropical Agriculture and Foresty, Hainan University, Hainan, China

**Keywords:** *sfp*, *lpaH2*, Antimicrobial activity, Phosphopantetheinyl transferase

## Abstract

**Background:**

*Bacillus* spp. have prominent ability to suppress plant pathogens and corresponding diseases. Previous analyses of *Bacillus* spp. revealed numerous gene clusters involved in nonribosomal synthesis of cyclic lipopeptides with distinct antimicrobial action. The 4′-phosphopantetheinyl transferase (PPTase) encoded by *sfp* gene is a key factor in lipopeptide synthesis in *Bacillus* spp. In previous study, *B. amyloliquefaciens* strain HAB-2 was found to inhibit a broad range of plant pathogens, which was attributed to its secondary metabolite lipopeptide.

**Results:**

A *sfp* homologue *lpaH2* which encoded phosphopantetheinyl transferase but shared 71% sequence similarity was detected in strain HAB-2. Disruption of *lpaH2* gene resulted in losing the ability of strain HAB-2 to produce lipopeptide, as well as antifungal and hemolytic activities. When *lpaH2* replaced *sfp* gene of *B. subtilis* strain 168, a non-lipopeptide producer, the genetically engineered strain 168 could produced lipopeptides and recovered antifungal activity. Quantitative PCR assays indicated that, the expression level of *lpaH2* in *B. subtilis* 168 strain decrease to 0.27-fold compared that of the wild type *B. amyloliquefaciens* strain HAB-2.

**Conclusion:**

Few studies have reported about *lpa* gene which can replace *sfp* gene in the different species. Taken together, our study showed for the first time that *lpaH2* from *B. amyloliquefaciens* could replace *sfp* gene.

**Electronic supplementary material:**

The online version of this article (doi:10.1186/s12866-017-1134-z) contains supplementary material, which is available to authorized users.

## Background

*Bacillus* spp. such as *B. amyloliquefaciens*, and *B. subtilis*, have prominent ability to suppress plant pathogens and corresponding diseases [1]. Some *Bacillus* spp. strains have been commercialized for disease control [2]. These beneficial bacteria can produce a wide variety of bioactive secondary metabolites [3], the majority of which belong to lipopeptide family, including surfactin, iturin and fengycin [4]. These compounds play an important role in their antimicrobial activities as well as suppressing plant diseases [5]. Surfactin contributes in disturbing biofilm formation, and is endowed with antiviral and antimycoplasma activities for clinical applications [6–10]; iturin displays antifungal and hemolytic activities with limited antibacterial activities [11]; fengycin has strong inhibition against filamentous fungi [12]. More recently, much research has gone into establishing lipopeptide synthesis genes, and the complete genome sequences of *Bacillus* strains provide a powerful tool for finding the functional genes [13].

Depending on specific *Bacillus* strains, the regulation of lipopeptide production may vary [4]. *Bacillus* species are well-known for antibiotics peptides production which are the most important bioactive metabolites via a series of modules mega-enzymes called non-ribosomal peptide synthetases (NRPSs) [14, 15]. In *Bacillus*, the genes corresponding to biosynthesis of cyclic lipopeptides are assembled as large gene clusters, which encode large multifunctional enzyme complexes, or NRPSs [1]. To synthetize cyclic lipopeptides, NRPSs possess a multidomain structure and affect the ordered recognition, activation, and linking of amino acids by utilizing the thiotemplate mechanism [16]. NRPSs also require posttranslational modification to be functionally active by 4′-phosphopantetheinyl transferase (PPTase) [17], which is encoded by *sfp* gene. This can be confirmed by that the model system *B. subtilis* 168 has the entire gene clusters for synthesizing surfactin and fengycin. However, due to the absence of *sfp* gene, these two lipopeptides are not produced [18]. Another example is that disruption of *sfp* gene of *B. amyloliquefaciens* FZB42 abolished the production of lipopeptides [19]. Furthermore, a native *sfp* gene was transferred into *B. subtilis* 168, which could restore the production of fengycin and surfactin lipopeptides [20]. Roongsawang et al. demonstrated that *sfp* has broad substrate preferences that allow posttranslational modification of both peptidyl carrier protein in the nonribosomal peptide synthetases and the acyl carrier protein [21]. All of these evidences indicate that *sfp* is indispensable in synthesis of lipopeptides.

In the previous study, strain HAB-2 was isolated from the rhizosphere soil of cotton plant and it showed strong antifungal activity [22]. MALDI-TOF MS and PCR analysis showed that strain HAB-2 lacked *sfp* gene although it could produce surfactin and iturin lipopeptides. Accordingly, we determined the role of *lpaH2* gene from strain HAB-2 in *B. amyloliquefaciens* and *B. subtilis*, based on the deletion of *lpaH2* from strain HAB-2 and complementation of *lpaH2* into the wild-type *B. subtilis* 168.

## Methods

### Bacterial and fungal cultures

Test organisms and plasmids used in this study are listed in the Table 1. The strain HAB-2 was isolated in the author’s laboratory from cotton field soil in Xinjiang Province, China and was deposited in China Center for Type Culture Collection (CCTCC), with accession number of ID.CCTCC M 2015070. All the *Bacillus* spp. cultures were routinely cultivated at 28 °C in Luria Bertani broth (LB) for 48 h. *Escherichia coli* was routinely cultivated at 37 °C in LB broth for 24 h. Potato dextrose agar (PDA) was used to culture *Colletotrichum gloeosporioides*.

**Table 1:** Organisms and plasmids used in this study

Organisms and plasmids	Description	Source
*Escherichia coli* DH5α	SupE44 ∆ lacU 169 Ø 80 lacZ ∆ M15 hsdR17 recA1 gyrA96 thi-1 relAF^−^	Transgen Bio Inc.
*Bacillus amyloliquefaciens HAB-2*	*lpaH2* gene, wild type, produces lipopeptides	Collection of this lab
*Bacillus amyloliquefaciens* HAB△*lpa*	deficient in lipopeptides; Cm^r^, Amp^r^	This work
*Bacillus subtilis* 168	deficient in lipopeptides	Collection of this lab
*Bacillus subtilis* 168 *lpa*	*lpaH2* gene, can produce lipopeptides; Cm^r^, Amp^r^	This work
*Colletotrichum gloeosporioides*	Indicator strain, causative agent of mango anthracnose	Collection of this lab
pAD 43–25 plasmid	Shuttle vector carrying GFP; Cm^r^	Collection of this lab
pMD18-T plasmid	T-clone site vector; *lac*Z; Amp^r^	TaKaRa Bio Inc.

*Amp* ^*r*^ ampicillin resistance, *Cm* ^*r*^ chloramphenicol resistance, *GFP* green fluorescent protein

### Morphological observation and molecular identification

The morphology of *B. amyloliquefaciens* was observed using scanning electron microscope (SEM). Prior to the observation, the cells of strain HAB-2 were centrifuged and then prefixed with 2.5% glutaraldehyde. The treated bacterial cells were rinsed three times each for 10 min with 100 mM phosphate buffer, post fixed for 3 h in 1% osmium tetroxide, and dehydrated through ethanol gradient. The samples were coated with gold and analyzed on Hitachi S-3000 N SEM (Hitachi, Japan). Micrographs were taken at 10.0 kV [23].

The chromosomal DNA of strain HAB-2 was extracted using Bacterial Genomic DNA Extraction Kit (Tiangen Biochemical Science and Technology Co., LTD) according to the manufacturer’s protocol. The 16S rRNA gene was amplified using PCR, with primer pair 27F/1492R (27F: 5′-AGAGTTTGATCATGGCTCAG-3′; 1492R: 5′-GGTTACCTTGTTACFACTT-3′) using the following program: pre-denaturation at 94 °C for 5 min, 35 cycles of amplification at 94 °C for 1 min, 50 °C for 31 min, and 72 °C for 2 min, and a final extension at 72 °C for 10 min [24]. The obtained sequences were analyzed using BLAST program of NCBI. The sequence alignment (produced using Clustal W) was imported into the program MEGA5, and the phylogenetic tree was constructed using the neighbor-joining method with 1000 bootstrap replicates.

### In vitro assay of antagonism

*B. amyloliquefaciens* strain HAB-2 was assayed for antagonism against 17 strains of plant pathogenic fungi (Additional file 1: Table S1). These pathogenic fungi were transferred onto the center of a PDA plate, and four culture plugs of *B. amyloliquefaciens* strain HAB-2 were placed near the edge of the same plate with same distance each other. The co-inoculated cultures were incubated at 28 °C for 72 h. Antifungal activity was determined by measuring growth inhibition by HAB-2. The inhibitory rate of mycelial growth (%) = (AB-Aa)/AB × 100% where Aa is the diameter of fungal colony on the line between fungal and bacterial inoculation pints, and AB is the diameter of fungal colony without bacterial inoculation. Each treatment had three replicates.

### Bioassay analysis of lipopeptides from *B. amyloliquefaciens* strain HAB-2

*B. amyloliquefaciens* strain HAB-2 was cultured in LB broth at 28 °C for 48 h under 170 rpm shaking. The culture medium was reduced in vacuo at 55 °C, and metabolites in the concentrated medium were extracted with n-butyl alcohol three times at 28 °C. The combined extracts were then concentrated in vacuo to dryness. The antifungal activity of lipopeptides was tested against *C. gloeosporioides* using paper disc method. *C. gloeosporioides* was inoculated into carboxymethylcellulose sodium (CMC) medium and grown for 3 days at 28 °C. The fermented liquid then filtered through 4 consecutive sterile absorbent cotton wool plugs to remove any hyphal fragments. The number of spores was counted using a hemocytometer, diluted to 10^6^ spores/mL. An aliquot of 4 mg/L lipopeptides was impregnated on a sterile filter paper discs (6 mm diameter), aseptically applied to the surface of an agar plate. Then the diameters of inhibition zones were measured after 48 h. The experiment was performed in triplicate.

### Chemical analysis of lipopeptides from strain HAB-2

Matrix-assisted laser desorption ionization–time of flight (MALDI-TOF) mass spectra was employed to analyze lipopeptides in HAB-2. The culture filtrate of HAB-2 was acidified to pH 2 with 6 N HCl and stored at 4 °C overnight, and then centrifuged. The precipitate was dissolved in methanol and filtered through a 0.2 μm polytetrafluoroethylene membrane. The experiment was conducted by means of a Bruker Daltonics Reflex MALDI-TOF mass spectrometer with a Scout-mtp ion source containing a 337 nm nitrogen laser. All spectra were acquired in the reflector positive ion mode. The acceleration and reflector voltages were 25 kV and 26.3 kV respectively. The matrix medium was a saturated solution of a-cyano-4-hydroxycinnamic acid in 30% aqueous acetonitrile containing 0.1% trifluoroacetic acid (*v*/v). For MALDI-TOF analysis, 1 μL extract was added onto the target plate, with 1 μL working solution, and then air dried.

### Transformation and DNA manipulation

Plasmids were extracted and purified using Omega Plasmid Miniprep Kit (Omega Bio-Tek, Norcros, GA, USA) following the manufacturer’s protocol. The genomic DNA was isolated using the Bacterial Genomic DNA Extraction Kit (Tiangen Biochemical Science and Technology Co., Ltd., Beijing, China) according to the manufacturer’s protocol. *E. coli* cell transformations were performed as described by Sambrook [25]; *B. subtilis* and *B. amyloliquefaciens* cells were transformed according to the method of Anagnostopoulos and Spizizen [26].

### Detection of lipopeptide and nonribosomal peptide synthetase genes

The synthetase and regulatory genes of lipopeptides including *ituC*, *srfAB*, *sboA*, *ituD*, *qk*, *fenD*, *bamC*, *yndj*, *ituB*, *fenB*, *ituA*, *lpa* and *sfp* (Additional file 1: Table S2), were amplified by PCR using specific primers which were designed on the basis of published genome sequences of *B. subillis* 168 (accession number CP019662.1), *B. subillis* RB14 (accession number D21876.1), and *B. amyloliquefaciens* FZB42 (accession number CP000560.1).

### Southern-blot hybridization

To confirm whether strain HAB-2 and 168 have *lpa* and *sfp* genes, Southern-blot hybridization analysis was carried out. The chromosomal DNA of strains HAB-2 and 168 were digested by *EcoR*Iand *Rsa*Ienzymes, then resolved on a 1% agarose gel by electrophoresis and transferred to a positively charged Nylon membrane (Roche Molecular Biochemicals, Mannheim, Germany). Southern-blot analysis was performed using the digoxigenin (DIG) DNA Labeling and Detection Kit (Roche Molecular Biochemicals, Germany) with *sfp* and *lpa* genes as a probe, according to the manufacturer’s protocol.

### Mutants and revertants construction

Recombination flanks were amplified by PCR. The upstream and downstream flanks were obtained using chromosomal DNA of *B. amyloliquefaciens* HAB-2 with the primer pairs F1/F2 and F5/F6, respectively (Additional file 1: Table S3). The chloramphenicol resistance gene fragment was amplified from P43–25 plasmid using with primers F3/F4 (Additional file 1: Table S3) by filling in the overlaps. The two gel-purified fragments were linked by Cmr resistance cassette and then cloned into plasmid pMD-18. The plasmid pMD18-T was transformed into the competent cells of strain HAB-2 and cells were plated on LB agar plates containing chloramphenicol, which were identified by testing for sensitivity against chloramphenicol. There were 176 mutants screened and isolated on a LB agar plate containing ampicillin and chloramphenicol, and transgenic positive mutants were verification by PCR with primers JD1 and JD2 (Additional file 1: Table S3).

To determine the effect of *lpaH2* gene on lipopeptide synthesis, *lpaH2* and chloramphenicol-resistant cassette were transformed into wild-type *B. subtilis* 168. For complementation analysis of *lpaHAB-2* in *B. sublilis* 168, the recombination flank fragments were amplified by PCR with the primer pairs J1/J2 and J7/J8, respectively (Additional file 1: Table S3). Amplification of *lpaH2* gene was carried out with primers J3/J4 and chloramphenicol gene from carrier plasmid (P43–25) was amplified with primers J5/J6 (Additional file 1: Table S3). Using fusion PCR, these fragments were linked and cloned into the vector pMD18-T and were transformed into the competent cells of *B. subtilis* 168 with the integration vector pMD18-T. The live mutants were isolated on a LB agar plate containing ampicillin and chloramphenicol, and transgenic positive mutants were verification with primers JD3 and JD4 (Additional file 1: Table S3).

To analyze the phenotype, spore suspension of *C. gloeosporioides* was spread on agar plate, followed by placing bacterial colony on the plate. Antifungal activities of bacterial strains were determined by measuring the inhibiting zone on *C. gloeosporioides*. The hemolysis activity of bacteria was tested on a blood agar plate.

### RNA extraction and qRT-PCR

The primers were designed by using Primer Premier 5.0 based on the sequence of *lpaH2* (GenBank: KX592168.1). Primer specificity was confirmed by agarose gel electrophoresis and melting curve analysis. The expression of *lpaH2* gene was monitored with qRT-PCR. The *16S rRNA* gene was used as the internal reference. The gene primers were listed in Additional file 1: Table S2. The 168*lpa* mutants and the wild type strain HAB-2 as control were cultured for 48 h. The RNAs were extracted following the RNAprep prue KIT (Tiangen) and complementary DNA (cDNA) was synthesized with the Prime Script RT-PCR kit (TaKaRa, Japan). The qRT-PCR assay used SYBR Premix EX Taq kit (TaKaRa) in 20 μL on the ABI Prism 7500 system. The program consisted of a Hot-Start activation step at 95 °C for 14 s, followed by 40 cycles of: 95 °C for 15 s, 56 °C for 30 s, 72 °C for 30 s. The experiment was repeated three times and the data were normalized according to the 2^-△△CT^ method.

## Results

### Morphology and identification of *B. amyloliquefaciens* HAB-2

**Fig. 1:**
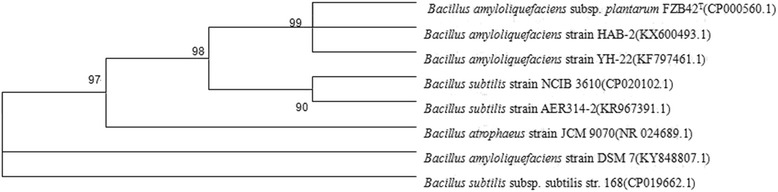
Phylogenetic tree constructed based on 16S rRNA sequences of HAB-2 and *Bacillus* spp. (16S rRNA sequences of strains retrieved from the NCBI GenBank. Bootstrap values were expressed as % of repetitions at branching points)

The cells of strain HAB-2 were intact, plump, and rod-shaped (Additional file 1: Figure S1). Sequence alignment showed that 16S rRNA gene of HAB-2 shared 99% similarity with that of *B. amyloliquefaciens* strain YH-22 (GenBank accession number KF797461.1) and 99% similarity with *B. amyloliquefaciens* subsp. *plantarum* FZB42^T^ (GenBank accession number CP000560.1) (Fig. 1). Based on these results, the strain HAB-2 was preliminary identified as *B. amyloliquefaciens* (GenBank accession number KX600493).

### Analysis of lipopeptides, their antifungal acitivites, and related genes in HAB-2

**Fig. 2:**
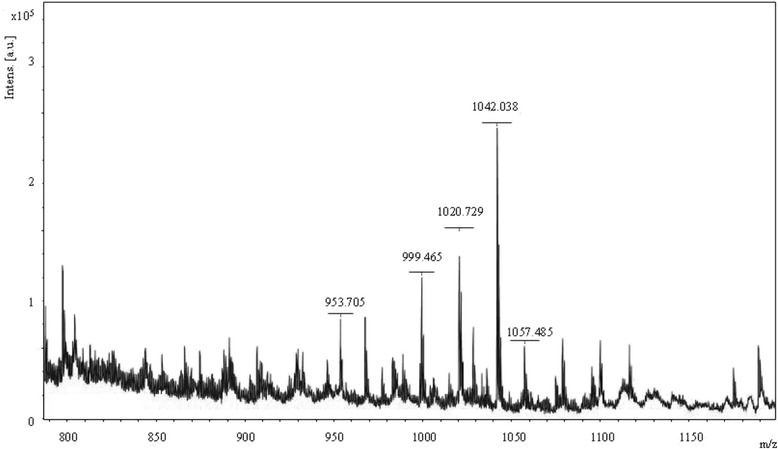
Chromatography of MALDI-TOF MS detecting lipopeptides in *B. amyloliquefaciens* strain HAB-2

*B. amyloliquefaciens* HAB-2 has antifungal activity to inhibit the growth of all test organisms with inhibition rates ranging from 42.99 to 69.76% on agar plates (Additional file 1: Table S3). The MALDI-TOF MS analysis is a convenient and efficient way to monitor qualitative results for lipopeptides in crude extracts which yielded complementary result. Using MALDI-TOF MS, several groups of mass signals were observed with the peaks at m/z = 928.868, 953.705, 999.465, 1020.729, 1042.038, 1057.485 and 1116.515 (Fig. 2 and Additional file 1: Table S4) [27, 28]. The result suggested that these peaks could be attributed to the isoform ensembles of surfactins and iturins [29], which provided a theoretical basis for the following studies.

Genes associated with the biosynthesis of lipopeptides in strain HAB-2 were cloned and sequenced. Genes such as *srfAB* (segment of 308 bp), *ituD* (647 bp), *fenD* (293 bp), *bamC* (850 bp), *yndj* (212 bp), *ituB* (508 bp) and *ituA* (885 bp) from HAB-2 could be amplified and had high degree of similarity to homologous sequences (Additional file 1: Figure S2), ranging from 97 to 99% (NCBI GenBank accession number DQ977668.1, HQ711614.1, DQ977668.1, CP006890.1, DQ977671.1, JN093026.1 and EU263005, respectively). *ituC*, *qk*, *fenB* genes could not amplified from strain HAB-2. This suggested that HAB-2 had the potential of producing lipopeptides including surfactins, fengycins, iturins, and bacillomycins.

**Fig. 3:**
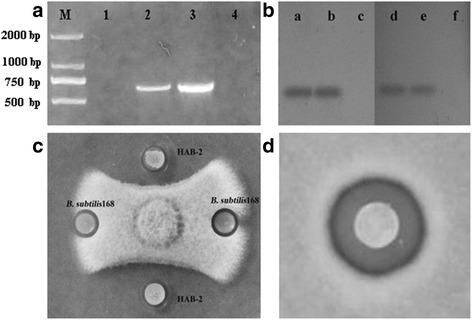
Lipopeptide analysis from *B. amyloliquefaciens* HAB-2 and *B. subtilis* 168. **a** Amplification of *lpa* and *sfp* gene fragments from *B. amyloliquefaciens* HAB-2 and *B. subtilis* 168. From lane 1 to 4: HAB-2-*sfp*; *B. subtilis* 168-*sfp*; HAB-2-*lpa*; and *B. subtilis* 168-*lpa*. Lane M: 2000 bp DNA marker. **b** Southern-blot hybridization for *lpaH2* and *sfp* genes, a: *lpa* from the strain HAB-2, b: chromosomal DNA of the strain HAB-2, c: chromosomal DNA of *B. subtilis* 168, d: *sfp* from *B. subtilis* 168, e: chromosomal DNA of *B. subtilis* 168, f: chromosomal DNA of the strain HAB-2. **c** Antifungal activity of *B. amyloliquefaciens* HAB-2 and *B. subtilis* 168 against *C. gloeosporioides* on agar plate. **d** Inhibitory effect of lipopetides produced from *B. amyloliquefaciens* HAB-2 against *C. gloeosporioides*

**Fig. 4:**
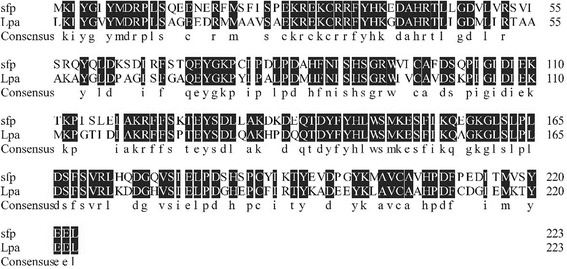
Amino acid sequence alignments of *lpa* in *B. amyloliquefaciens* HAB-2 and *sfp* of *B. subtilis* 168. (Highlighted letters indicate identity)

Only *lpa* gene (675 bp) was detected in strain HAB-2, in contrast, *sfp* gene (675 bp) was amplified in strain 168 (Fig. 3a). Sequence analysis indicated that *lpa* fragment from strain HAB-2 shared 99% similarity with *B. subtilis* RB14 (D21876.1). This fragment was accordingly named as *lpaH2* (Accession KX592168) in strain HAB-2. A panel in Fig. 3b shows the signal for the *lpa* fragment in strain HAB-2. As expected, one signal was identified for the *sfp* gene fragment in strain 168. The southern hybridization results demonstrated that strain HAB-2 genome contains *lpa* gene but lacks *sfp* gene (Fig. 3c). Bioassays showed that *sfp-*inactive strain *B. subtilis* 168 did not inhibit the growth of *C. gloeosporioides* but strain HAB-2 did (Fig. 3d). HAB-2-derived lipopeptides at the concentration of 4 mg/L exhibited an antifungal activity with an inhibitory zone of 12.2 ± 0.1 mm (Fig. 3e). Lpa family demonstrated high homology and a similar organization to Sfp family which encoded 4′-phosphopantetheinyl transferase [30]. The *lpa* gene of strain HAB-2 shared 71% amino acid identity with *sfp* of *B. subtilis* 168 (Fig. 4) Generally, *sfp* is recognized as essential element for activation of lipopeptides formation. Therefore, we proposed that *lpa* of strain HAB-2 plays critical roles in lipopeptides formation [19].

### Examining the biological function of *lpaH2* gene by deletion

**Fig. 5:**
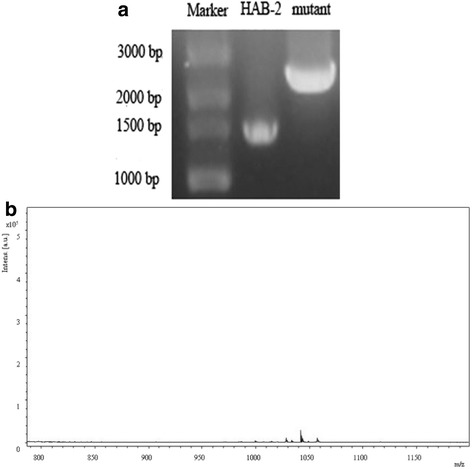
Characterization of *B. amyloliquefaciens* strain HAB-2 and its mutant. **a** Gel electrophoresis of PCR products of wild-type strain HAB-2 (lane HAB-2) and its mutant (lane Mutant); **b** MALDI-TOF MS analysis on the extract of HAB△*lpa* mutant, 3000 bp DNA marker

The plasmid pMT-18 was constructed for deletion of *lpa* gene from strain HAB-2. Double crossover homologous recombination resulted in the deletion of a 0.6-kb *lpa* fragment and was replacement with chloramphenicol (Cmr)-resistance cassette on the chromosome. Two primers amplified the segment containing the genes to facilitate identification of positive transformants and Cmr transformants were selected. Disruption of *lpaH2* derived from the strain HAB-2 was documented. The transformation resulted in mutants carrying plasmid pMT-18 (Fig. 5a). The fragment linked Cm-resistant and *lpaH2* genes (2.5 kb in size) were detected. The effect of the HAB△*lpa* mutant on lipopeptide synthesis was monitored by growth inhibition of *C. gloeosporioides*. The results demonstrated that wild-type strain HAB-2 has inhibition ability, in the contrast antimicrobial actions of the HAB△*lpa* mutants were abolished (Additional file 1: Figure S3A and B). Compared to the wild-type strain HAB-2, which had ability to lyse the blood cells by surface-active compounds and cause a halo on blood agar plate, the hemolytic activities of HAB-2 derivatives in the absence of *lpaH2* were also abolished based on the observation of no halo (Additional file 1: Figure S3C and D). It suggests that strain HAB-2 derivatives in the absence of *lpaH2* could not produce surfactin. Mass spectra data suggested that strain HAB-2 could produce lipopetides including iturin and surfactin (Fig. 2). However, the HAB△*lpa* mutant had complete deficiency of lipopeptide production (Fig. 5b).

### Examining the biological function of *lpaH2* gene by complementation

**Fig. 6:**
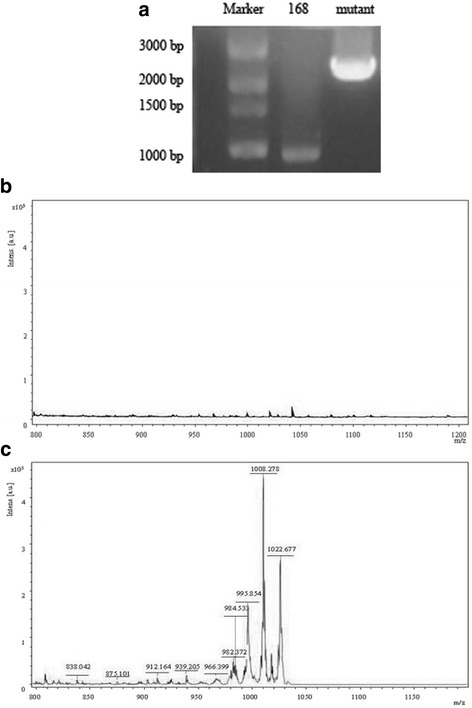
Characterization of wild-type *Bacillus subtilis* stain 168 and its mutant. **a** Gel electrophoresis of PCR products of wild-type strain 168 (lane 168) and its mutant (lane Mutant); **b** MALDI-TOF-MS analysis on extracts of wild-type strain 168; **c** MALDI-TOF-MS analysis on the extract of strain 168 mutant, 3000 bp DNA marker

The *lpaH2* gene was successfully integrated into the chromosome of *B. subtilis* 168 by homologous recombination (Fig. 6). The mutants have antifungal activity against *C. gloeosporioides* (Additional file 1: Figure S4A and B). Hemolytic activity of the 168lpa mutant was detected on blood agar plate (Additional file 1: Figure S4C and D), which was an evidence for surfactin production. Mass spectrum showed peaks at m/z values 1008 and 1022, which could be attributed to the surfactin of the extract of the 168lpa mutant (Fig. 6b and c).

### Effect on *lpaH2* gene expression

**Fig. 7:**
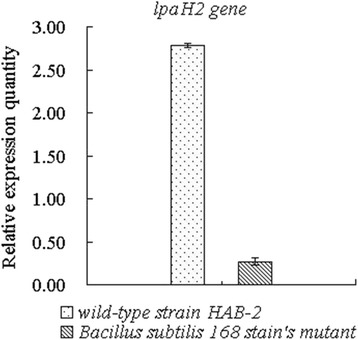
The qRT-PCR analysis of expression of lpaH2 in *B. subtilis* 168 stain’s mutant and wild-type strain HAB-2 at 48 h. The values were normalized to the levels of 16S rRNA, which is a housekeeping gene as an internal reference gene. The y-axis values represent the mean expression ± the standard deviations (*n* ≥ 3) relative to the control

Quantitative PCR assays indicated that the *lpaH2* integrated into the *B .subtilis* 168 strain makes the expression level to decrease to 0.27-fold of the wild type *B. amyloliquefaciens* strain HAB-2 (Fig. 7).

## Discussion

We have demonstrated that strain HAB-2 was most closely related to *B. amyloliquefaciens* subsp. *plantarum* FZB42^T^. Based on the production of liquefying amylase, *B. amyloliquefaciens* was added as its own distinct species in 1987 [31]. *B. amyloliquefaciens* subsp. *plantarum* was in the clade of *B. amyloliquefaciens* and the genome of type strain FZB42 was revealed in 2007 [13]. However, recent research reported that *B. amyloliquefaciens* subsp. *plantarum* should be reclassified as later heterotypic synonyms of *B. methylotrophicus* KACC 13015^T^ or *B. velezensis* NRRL B-41580 T [32, 33].

*B. amyloliquefaciens* HAB-2 had inhibitory activity against 17 plant pathogenic fungi when 7 lipopeptide synthetase genes, including *ituB*, *srfAB*, *ituD*, *fenD*, *yndj*, *ituA*, *bamC*, were cloned from strain HAB-2. These lipopeptides of strain HAB-2 belonged to surfactin, iturins and fengycin families. As mentioned above, *sfp* encodes PPTase which plays an essential role in priming synthases, NRPS, from inactive apo forms to active holo forms [19, 34, 35]. The non-lipopeptide-producer *B. subtilis* 168 has the peptide synthetase [36], due to a frameshift mutation in *sfp* responsible for conversion of nascent antibiotic synthetases to active holoforms [11]. However, the strain HAB-2 also lacked *sfp* gene whereas lipopeptides biosynthesis remained unimpaired. It suggests that strain HAB-2 has biosynthetic machinery for different lipopeptides production. From the amino acid sequence analysis, *lpaH2* gene in the strain HAB-2 was identified and was similar to the *sfp* gene. Some reports indicated that *lpa* gene showed 73% homology with *sfp* and is crucial to the regulation of surfactin and iturin A production in *B. subtilis* or *B. pumilus* [30, 37, 38]. Tsuge et al. demonstrated that *lpa-8* is a regulatory protein that acts as a transcription initiation factor and is required for amino acid transfer, for peptide secretion, or for the transport system of the peptides [39]. Mutational analysis of *lpaH2* and the effect on corresponding lipopeptides production aided us in elucidating the function of *lpaH2*. We hypotheze that *lpaH2* plays a similar role with *sfp* gene in the synthesis of lipopeptides in HAB-2 strain. Thus, *lpaH2* gene was knocked out in strain HAB-2 by double crossover homologous recombination with antibiotic and hemolytic activities abolished, as observed in *B. subtilis* 168. Our results also seem to support that *lpaH2* has functional homology with *sfp* gene. We tentatively conclude that *lpaH2* is responsible for activating lipopeptide synthases, which replaces the function of *sfp* in strain HAB-2.

*Sfp* synthesizes bioactive peptides in plant-dependent pathways of secondary metabolism of *Bacillus* spp. Li et al. [24] demonstrated that *B. subtilis* 168 genetically engineered with *sfp* gene synthesized a large amount of surfactin. A similar observation has been reported that *B. subtilis* 168 derivatives introduced an intact copy of *sfp* recovered production of polyketide bacillaene [40]. Furthermore, *lpa-14* showed 72% homology with *sfp*, which is responsible for surfactin in *B. subtilis* RB14 convert *B. subtilis* 168 to a surfactin-producer [38, 41]. Tsuge et al. [20] revealed that *B. subtilis* 168 expressing only *lpa-8* can produce plipastatin. Nevertheless, *B. subtilis* 168 by transformation of *lpaB3,* isolated from *B. subtilis* B3, could not produce iturin A [42]. It was suggested that *lpaB3* gene possibly regulated biosynthesis surfactin in *B. subtilis* strain 168 [42], which explain *lpa* gene regulation varies in peptide antibiotics uniqueness among *Bacillus* species [38]. However, there have been few studies reported on *lpa* gene which can replace *sfp* gene, especially between the *B. subtilis* and *B. amyloliquefaciens* strains. When *lpaH2* replaced *sfp* gene of *B. subtilis* strain 168, a non-lipopeptide producer, the genetically engineered strain 168 possessed antagonistic and hemolysis properties. Our study is the first time to investigate that *lpaH2* from *B. amyloliquefaciens* could replace *sfp* gene and also played a major role in *B. subtilis*. Besides this, the inhibition of mutant has not increase significantly, which might be due to the transformation of the exogenous gene from *B. amyloliquefaciens* strain into *B. subtilis*. We suggest that it might be partly explained by the different similarity of *lpaH2* and *lpa* with *sfp*, which is 72% or 73% homology with *sfp* in the previous reports. The antifungal ability can be enhanced by increasing the strong promoter in subsequent experiments.

## Conclusions

*lpaH2* was equally important as *sfp* gene in lipopeptide synthesis in *B. amyloliquefaciens* HAB-2. Our current work investigating the functions and relationships between Lpa and Sfp families will help to understand the evolutionary and genetic characteristics of these genes. Hence, further work will be necessary to understand that evolutive and genetical characteristics in Lpa family and Sfp family, individually or in combination, contributed to productions.

## Additional file

Additional file 1: Characterization of *lpaH2* gene corresponding to lipopeptide synthesis in *Bacillus amyloliquefaciens* HAB-2. (PDF 1085 kb)

## Abbreviations

16S rRNA: 16S ribosomal RNA
Ampr: Ampicillin resistance
CCTCC: China Center for Type Culture Collection
CMC: Carboxymethylcellulose sodium
Cmr: Chloramphenicol resistance
DIG: Digoxigenin DNA labeling
GFP: Green fluorescent protein
LB: Luria-Bertan medium
*lpaH2*: Encoded phosphopantetheinyl transferase
MALDI-TOF: Matrix-assisted laser desorption ionization-time of flight
NRPS: Nonribosomal peptide synthetases
PCR: Polymerase chain reaction
PDA: Potato dextrose agar
PPTase: 4′-phosphopantetheinyl transferase
SEM: Scanning electron microscope
*Sfp*: Encoded phosphopantetheinyl transferase

## References

[1] Ongena M, Jacques P (2008). *Bacillus* lipopeptides: versatile weapons for plant disease biocontrol. Trends Microbiol.

[2] Wu L, Wu H, Chen L, Lin L, Borriss R, Gao X (2015). Bacilysin overproduction in *Bacillus amyloliquefaciens* FZB42 markerless derivative strains FZBREP and FZBSPA enhances antibacterial activity. Appl Microbiol Biotechnol.

[3] Stein T (2005). *Bacillus subtilis* antibiotics: structures, syntheses and specific functions. Mol Microbiol.

[4] Kumar A, Sani S, Wray V, Nimtz M, Prakash A, Johri B (2012). Characterization of an antifungal compound produced by *Bacillus sp*. strain A_5_F that inhibits *Sclerotinia sclerotiorum*. J Basic Microbiol.

[5] Chowdhury S, Uhl J, Grosch R, Alqueres S, Pittroff S, Dietel K, Schmitt K, Borriss R, Hartmann A (2015). Cyclic lipopeptides of *Bacillus amyloliquefaciens* subsp. plantarum colonizing the lettuce rhizosphere enhance plant defense responses toward the bottom rot pathogen rhizoctonia solani. Mol Plant Microbe In.

[6] Magnet-Dana R, Peypoux F (1994). Iturins, a special class of pore forming lipopeptides: biological and physicochemical properties. Toxicology.

[7] Vollenbroich D, Özel M, Vater J, Kamp R, Pauli G (1997). Mechanism of inactivation of enveloped viruses by the biosurfactant surfactin from *Bacillus subtilis*. Biologicals.

[8] Vollenbroich D, Pauli G, Ozel M, Vater J (1997). Antimycoplasma properties and application in cell culture of surfactin, a lipopeptide antibiotic from *Bacillus subtilis*. Appl Environ Microb.

[9] Seydlová G, Svobodová J (2008). Review of surfactin chemical properties and the potential biomedical applications. Cent Eur J Med.

[10] Tabbene O, Kalai L, Slimene I, Karkouch I, Elkahoui S, Gharboi A, Cosette P, Mangoni M, Jouenne T, Limam F (2011). Anti-Candida effect bacillomycin D-like lipopeptides from *Bacillus subtilis* B38. FEMS Microbiol Lett.

[11] Romero D, de Vicente A, Rakotoaly R, Dufour S, Veening J, Arrebola E, Cazorla F, Kuipers O, Paquot M, Pérez-García A (2007). The iturin and fungycin families of lipopeptides are key factors in antagonism of *Bacillus subtilis* towards *Podosphaera fusca*. Mol Plant Microbe In.

[12] Grover M, Nain L, Singh B, Saxena A (2009). Molecular and biochemical approaches for characterization of antifungal trait of a potent biocontrol agent *Bacillus subtilis* RP24. Curr Microbiol.

[13] Chen X, Koumoutsi A, Scholz R, Eisenreich A, Schneider K, Heinemeyer I, Morgenstern B, Voss B, Hess W, Reva O, Junge H, Voigt B, Jungblut P, Vater J, Süssmuth R, Liesegang H, Strittmatter A, Gottschalk G, Borriss R (2007). Comparative analysis of the complete genome sequence of the plant growth-promoting bacterium *Bacillus amyloliquefaciens* FZB42. Nat Biotechnol.

[14] Mukherjee A, Das K (2005). Correlation between diverse cyclic lipopeptides production and regulation of growth and substrate utilization by *Bacillus subtilis* strains in a particular habitat. FEMS Microbiol Ecol.

[15] Ongena M, Jourdan E, Adam A, Paquot M, Brans A, Joris B, Arpigny J, Thonart P (2007). Surfactin and fengycin lipopeptides of *Bacillus subtilis* as elicitors of induced systemic resistance in plants. Environ Microbiol.

[16] Walsh C (2004). Polyketide and nonribosomal peptide antibiotics: modularity and versatility. Science.

[17] Koumoutsi A, Chen XH, Vater J, Borriss R (2007). *DegU* and *YczE* positively regulate the synthesis of bacillomycin D by *Bacillus amyloliquefaciens* strain FZB42. Appl Environ Microb.

[18] Koumoutsi A, Chen X, Henne A, Liesegang H, Hitzeroth G, Franke P, Vater J, Borriss R (2004). Structural and functional characterization of gene clusters directing nonribosomal synthesis of bioactive cyclic lipopeptides in *Bacillus amyloliquefaciens* strain FZB42. J Bacteriol.

[19] Chen X, Koumoutsi A, Scholz R, Borriss R (2009). More than anticipated-production of antibiotics and other secondary metabolites by *Bacillus amyloliquefaciens* FZB42. J Mol Microb Biotech.

[20] Tsuge K, Ano T, Hirai M, Nakamura Y, Shoda A (1999). The genes *degQ*, *pps* and *lpa-8* (*sfp*) are responsible for conversion of *Bacillus subtilis* 168 to plipastatin production. Antimicrob Agents Ch.

[21] Roongsawang N, Thaniyavarn J, Thaniyavarn S, Kameyama T, Haruki M, Imanaka T, Morikawa M, Kanaya S (2002). Isolation and characterization of a halotolerant *Bacillus subtilis* BBK-1 which produces three kinds of lipopeptides: bacillomycin L, plipastatin, and surfactin. Extremophiles.

[22] Jin P, Wang H, Liu W, Fan Y, Miao W. A new cyclic lipopeptide isolated from *Bacillus amyloliquefaciens* HAB-2 and safety evalution. Pestic Biochem Physiol. 2017; doi:10.1016/j.pestbp.2017.08.015. 10.1016/j.pestbp.2017.08.015 29933991

[23] Wu L, Wu H, Chen L, Yu X, Borriss R, Gao X (2015). Difficidin and bacilysin from *Bacillus amyloliquefaciens* FZB42 have antibacterial activity against *Xanthomonas oryzae* rice pathogens. Sci Rep.

[24] Li X, Wu H, Zhang Y, Lu Q, Gao X (2015). Function of *degQ* and *sfp* and their effects on fengycin productivity of *Bacillus subtilis*. Chin J Biol Control.

[25] Sambrook J, Fritsch E, Maniatis T (1982). Molecular cloning: a laboratory manual. Cold Spring Harbor laboratory press. Cold Spring Harbor.

[26] Anagnostopoulos C, Spizizen J (1961). Requirements for transformation in *Bacillus subtilis*. J Bacteriol.

[27] Vater J, Kablitz B, Wilde C, Franke P, Mehta N, Cameotra SS (2002). Matrix-assisted laser desorption ionization-time of flight mass spertrometry of lipopeptide biosurfactants in whole cells and culture filtrates of *Bacillus subtilis* C-1 isolated from petroleum sludge. Appl Environ Microbl.

[28] Pabel CT, Vater J, Wilde C, Franke P, Hofemeister J, Adler B, Bringmann G, Hacker J, Hentschel U (2003). Antimicrobial activities and matrix-assisted laser desorption/ionization mass spectrometry of *Bacillus* isolates from the marine sponge *Aplysina aerophoba*. Mar Biotechnol.

[29] Leenders F, Stein T, Kablitz B, Franke P, Vater J (1999). Rapid typing of *Bacillus subtilis* strains by their secondary metabolites using matrix-assisted laser desorption/ionization mass spectrometry of intact cells. Rapid Commun Mass Sp.

[30] Yao S, Gao X, Fuchsbauer N, Hillen W, Vater J, Wang J (2003). Cloning, sequencing, and characterization of the genetic region relevant to biosynthesis of the lipopeptides iturin a and surfactin in *Bacillus subtilis*. Curr Microbiol.

[31] Priest F, Goodfellow M, Shute L, Berkeley W (1987). *Bacillus amyloliquefaciens* sp. nov., nom. rev. Int J Syst Bact.

[32] Dunlap C, Kim S, Kwon S, Rooney A (2015). Phylogenomic analysis shows that *Bacillus amyloliquefaciens* subsp. *plantarum* is a later heterotypic synonym of *Bacillus methylotrophicus*. Int J Syst Evol Microbiol.

[33] Dunlap C, Kim S, Kwon S, Rooney A (2016). *Bacillus velezensis* is not a later heterotypic synonym of *Bacillus amyloliquefaciens*; *Bacillus methylotrophicus*, *Bacillus amyloliquefaciens* subsp. *plantarum* and ‘*Bacillus oryzicola*’ are later heterotypic synonyms of *Bacillus velezensis* based on phylogenomics. Int J Syst Evol Microbiol.

[34] Lambalot R, Gehring A, Flugel R, Zuber P, LaCelle M, Marahiel M, Reid R, Khosla C, Walsh C (1996). A new enzyme superfamily: the phosphopantetheinyl transferases. Chem Biol.

[35] Beld J, Sonnenschein E, Vickery C, Noel J, Burkart M (2013). The Phosphopantetheinyl transferases: catalysis of a posttranslational modification crucial for life. Nat Prod Rep.

[36] Reuter K, Mofid M, Marahiel M, Ficner R (1990). Crystal structure of the surfactin synthetase-activating enzyme *sfp*: a prototype of the 4′-phosphopantetheinyltransferase superfamily. EMBO J.

[37] Nakano M, Marahiel M, Zuber P (1988). Identification of a genetic locus required for biosynthesis of the lipopeptide antibiotic surfactin in *Bacillus subtilis*. J Bacteriol.

[38] Huang C, Ano T, Shoda M (1993). Nucleotide sequence and characteristics of the gene, *lpa-14*, responsible for biosynthesis of the lipopeptide antibiotics iturin a and surfactin from *Bacillus subtilis* RB14. J Ferment Bioeng.

[39] Tsuge K, Ano T, Shoda A (1996). Isolation of a gene essential for biosynthesis of the lipopeptide antibiotics plipastatin B1 and surfactin in *Bacillus subtilis* YB8. Arch Microbiol.

[40] Chen X, Vater J, Piel J, Franke P, Scholz R, Schneider K, Koumoutsi A, Hitzeroth G, Grammel N, Strittmatter A, Gottschalk G, Süssmuth R, Borriss R (2006). Structural and functional characterization of three polyketide synthase gene clusters in *Bacillus amyloliquefaciens* FZB42. J Bacteriol.

[41] Hiraoka H, Ano T, Shoda M (1992). Molecular cloning of a gene responsible for the biosynthesis of the lipopeptide antibiotics iturin and surfactin. J Ferment Bioeng.

[42] Yao S, Gao X, Fuchsbauer N, Hillen W, Vater J, Wang J (2003). Identification of the genetic region of *Bacillus subtilis* B3 renders *Bacillus subtilis* 168 biosynthesis of lipopeptide surfactin positive. Biocontrol Sci Techn.

