# Supplementary material for: Characterization of lpaH2 gene corresponding to lipopeptide synthesis in Bacillus amyloliquefaciens HAB-2
Source: BMC Microbiol. 2017 Dec 4;17:227. doi: 10.1186/s12866-017-1134-z (PMC5716053; doi:10.1186/s12866-017-1134-z)
Supplement: Additional file 1: — Characterization of lpaH2 gene corresponding to lipopeptide synthesis in Bacillus amyloliquefaciens HAB-2. (PDF 1085 kb) [file 12866_2017_1134_MOESM1_ESM.pdf]

Characterization of *lpaH2* gene corresponding to lipopeptide synthesis in *Bacillus amyloliquefaciens* HAB-2

Pengfei Jin, Haonan Wang, Wenbo Liu, Weiguo Miao

Institute of Tropical Agriculture and Foresty, Hainan University, Haikou 570228, Hainan, China

Corresponding author Weiguo Miao: E-mail: miao@hainu.edu.cn

Figure. S1 Morphological observation of *Bacillus amyloliquefaciens* strain HAB-2 under scanning electron microscope. Bar = 1 μm.

Figure. S2 Gel electrophoresis of amplified segments of functional genes for lipopeptide biosynthesis in *B. amyloliquefaciens* strain HAB-2, using polymerase chain reaction. Bands are target genes on each lane. Lane 1: *ituC,* 2: *srfAB,* 3: *sboA,* 4: *ituD,* 5: *qk,* 6: *fend,* 7: *bamC,* 8: *yndj,* 9: *ituB,* 10: *fenB,* 11: *ituA,* and M: 2000 DNA marker.

Figure. S3 Effects of *B. amyloliquefaciens* strain HAB-2 and its mutants in antagonism and hemolytic activity. (A) Inhibitory effect of of strain HAB-2 against *C. gloeosporioides*; (B) Effect of HAB△*lpa* mutant against *C. gloeosporioides*; (C) Hemolytic activity of strain HAB-2 on a blood agar plate; (D) Hemolytic activity of the HAB△*lpa* mutant on a blood agar plate.

Figure. S4 Effects of *B. subtilis* stain 168 and its mutants in antagonism and hemolytic activity. (A) Inhibitory effect of strain 168 against *C. gloeosporioides*; (B) Inhibitory effect of the mutant of 168 against *C. gloeosporioides*; (C) Hemolytic activity of strain 168; (D) Hemolytic activity of the mutant of strain 168.

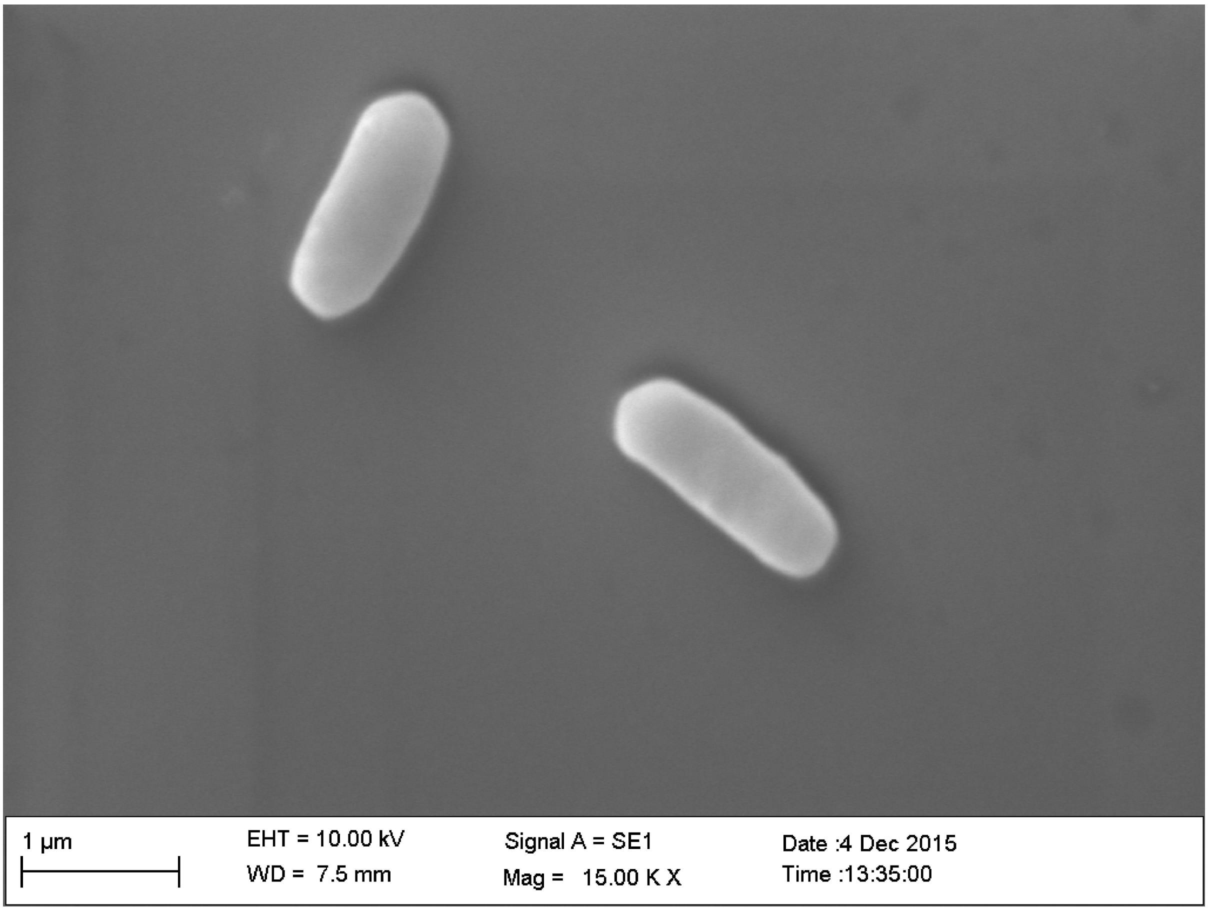

Fig. S1 Morphological observation of *B. amyloliquefaciens* strain HAB-2 under scanning electron microscope. Bar = 1 μm.

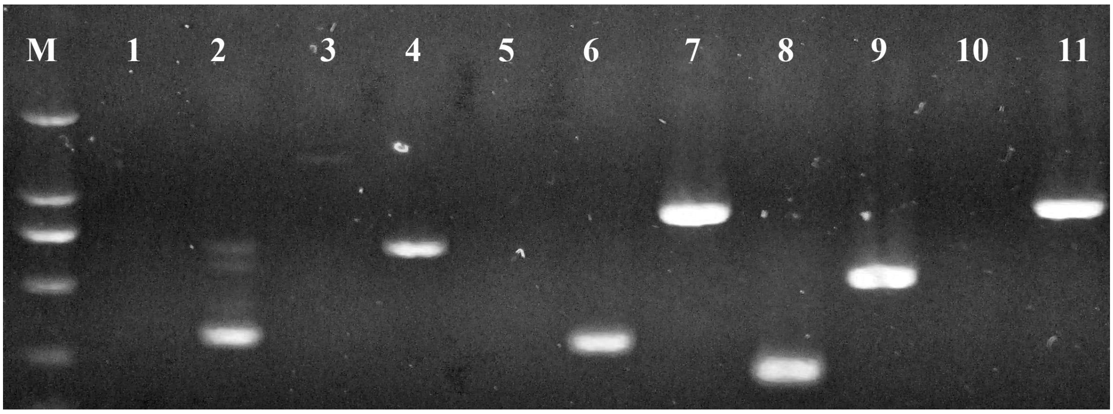

Fig. S2 Gel electrophoresis of amplified segments of functional genes for lipopeptide biosynthesis in *B. amyloliquefaciens* strain HAB-2.

Lane 1: *ituC,* 2: *srfAB,* 3: *sboA,* 4: *ituD,* 5: *qk,* 6: *fend,* 7: *bamC,* 8: *yndj,* 9: *ituB,* 10: *fenB,* 11: *ituA,* and M: 2000 DNA marker.Bands are target genes on each lane.

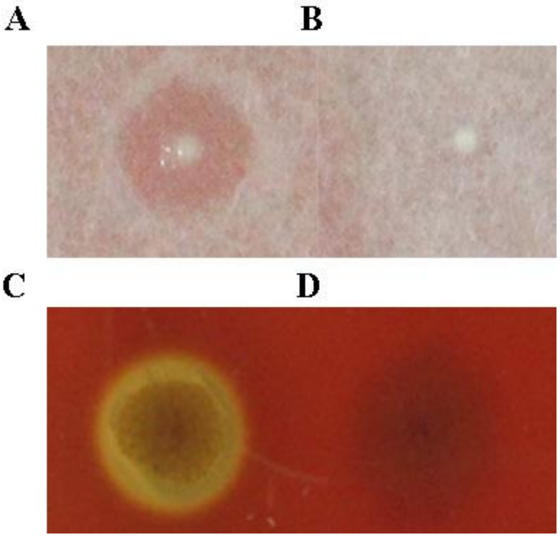

Fig. S3 Effects of *B. amyloliquefaciens* strain HAB-2 and its mutants in antagonism and hemolytic activity.

(A) Inhibitory effect of of strain HAB-2 against *C. gloeosporioides*; (B) Effect of HAB△*lpa* mutant against *C. gloeosporioides*; (C) Hemolytic activity of strain HAB-2 on a blood agar plate; (D) Hemolytic activity of the HAB△*lpa* mutant on a blood agar plate.

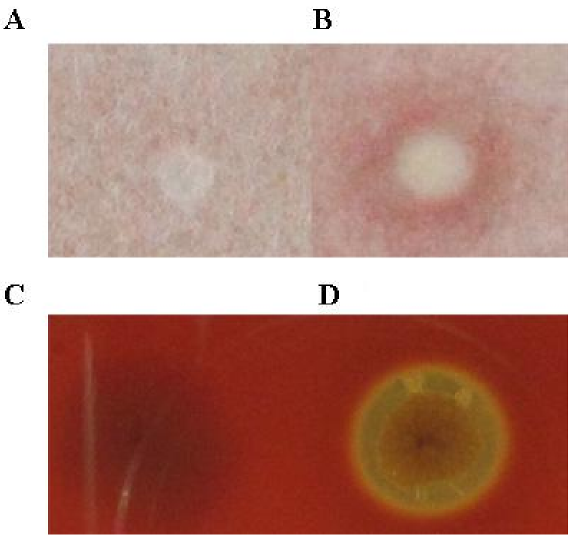

Fig. S4 Effects of *B. subtilis* stain 168 and its mutants in antagonism and hemolytic activity. (A) Inhibitory effect of strain 168 against *C. gloeosporioides*; (B) Inhibitory effect of the mutant of 168 against *C. gloeosporioides*; (C) Hemolytic activity of strain 168; (D) Hemolytic activity of the mutant of strain 168.

Table S1 Activity of *Bacillus amyloliquefaciens* strain HAB-2 against 17 strains of plant pathogens

Isolates	CK	HAB-2	
	Diameter of colony (mm）	Colony diameter (mm)	Inhibition rate（％）
Fusarium oxysporum f.sp.*cubense*, FOC (Dongfang, Hainan)	(61.60±0.05)	（23.98±1.54）	（61.07±2.50）FGHIefgh
Colletotrichum orbiculare (Wenchang, Hainan)	(67.65±0.63)	（28.34±1.66）	（58.12±2.46）EFGHefg
Colletotrichum orbiculare (Bark, &Mont.)V Arx (Haikou, Hainan)	(65.75±0.88)	（25.08±0.83）	（61.86±1.26）GHIJefgh
Fusarium oxysporum f.sp.*cubense*, FOC (Danzhou, Hainan)	(60.456±0.74)	（27.50±0.16）	（54.52±0.26）CDEcde
Fusarium oxysporum f.sp.*cubense*, FOC (Changjiang, Hainan)	(58.01±1.47)	（22.42±1.48）	（61.35±2.21）FGHIefgh
Corynespora cassiicola (Qongzhong, Hainan)	(59.63±0.15)	（22.53±1.29）	（62.22±2.17）HIJfgh
Colletotrichum gloeosporioides Penz (Qiongzhong, Hainan)	(68.66±0.94)	（30.77±1.05）	（55.19±1.52）DEFcdef
Gloeospotium psidii Delacr (Haikou, Hainan)	(68.70±0.75)	（22.10±1.40）	（67.83±2.05）JKhi
Fusarium oxysporum.sp.*cucumebrium* Owen (Dingan, Hainan)	(67.17±0.08)	（36.39±1.58）	（45.83±2.35）ABab
Fusarium oxysporum f.sp.*cubense*, FOC (Linshui, Hainan)	(60.04±0.54)	（34.23± 2.30）	（42.99±3.82）Aa
Dothiorella dominicana Cif (Danzhou, Hainan)	(57.38±0.11)	（17.35±0.03）	（69.76±0.06）Ki
Alternaria solani (E.et M.) Jones et Grout (Haikou, Hainan)	(51.86±1.69)	（18.79±1.06）	（63.77±2.04）HIJKghi
Colletotrichum gloeosporioides Penz (danzhou, Hainan)	(70.49±0.29)	（27.16±1.68）	（61.47±2.38） FGHIJefgh
FusaHum graminearum Sehw. (Zhengzhou, Henan)	(67.87±1.05)	（30.10±0.17）	（55.65±0.25）DEFGdef
Botryodiplodia theobromae Pat (Danzhou, Hainan)	(70.44±0.12)	（36.76±1.60）	（47.81±2.28）ABabc
Botryospuaeria berengeriana de Not .t .sp. *piricola* (Yantai, Shandong)	(70.82±0.49)	（29.58±1.89）	（58.24±2.66）EFGHefg
Fusarium oxysporum.sp. *cucumebrium* Owen (Wenchang, Hainan)	(66.18±0.53)	（32.75±1.11）	（50.51±1.68）BCDbcd

Values by the different capital letters indicated significantly different at $P < 0.05$; Values by

the different lowercase letters indicated significantly different at $P < 0.01$.

Table S2 Primers in this study

Gene	Sequence (5’-3’)	Thermal cycler setting
yndj	CAGAG CGACA GCAAT CACAT<br>TGAAT TTCGC TCCGC TTATC	95 °C for 5 min, 30 cycles of 95 °C for 60 s, 52 °C for 30 s, 72 °C for 60 s; 72 °C for 10 min
bamC	CTGGA AGAGA TGCCG CTTAC<br>AAGAG TGCGT TTTCT TCGGA	
qk	CTTAA ACGTC AGAGG CGGAG<br>ATTGT GCAGC TGCTT GTACG	
sboA	TCGGT TTGTA AACTT CAACT GC<br>GTCCA CTAGA CAAGC GGCTC	
ituC	TTCAC TTTTG ATCTG GCGAT<br>CGTCC GGTAC ATTTT CAC	
fenD	CCTGC AGAAG GAGAA GTGAAG<br>TGCTC ATCGT CTTCC GTTTC	
fenB	TACCT ATCGC AATGT CGTGT<br>CTTCG ATTTC TAACA GCCGC	
ituB	AAGAA GGCGT TTTTC AAGCA<br>CGACA TACAG TTCTC CCGGT	
ituD	GATGC GATCT CCTTG GATGT<br>ATCGT CATGT GCTGC TTGAG	
ituA	TGCCA GACAG TATGA GGCAG<br>CATGC CGTAT CCACT GTGAC	
srfAB	GTTCT CGCAG TCCAG CAGAAG<br>GCCGA GCGTA TCCGT ACCGAG	
lpa	GTTCT CGCAG TCCAG CAGAAG<br>GCCGA GCGTA TCCGT ACCGAG	94 °C for 5 min; 30 cycles of 94 °C for 1 min; 58 °C for 30 s; 72 °C for 1 min 30 s; 72 °C for 10 min
sfp	GTTCT CGCAG TCCAG CAGAAG<br>GCCGA GCGTA TCCGT ACCGAG	94 °C for 3 min; 30 cycles of 94 °C for 1 min; 43°C for 1 min; 72 °C for 1 min; 72 °C for 10 min

16S rRNA	TTACTCTGCGGCTGCTTCT<br>ATGCGCCATCGTAAGAGTG	94 °C for 5 min; 30 cycles of 94 °C for 1 min; 49°C for 1 min; 72 °C for 1 min; 72 °C for 10 min

Table S3 Primers in this study

Gene	Sequence (5'-3')	Thermal cycler setting
F1	AAAAACGAGAGCTTGTCCGA	95 °C for 5 min, 30 cycles of 95 °C for 1 min, 55°C for 30 s, 72°C for 1 min; 72°C for 10 min
F2	TCAAAGCCTTGTGTATCACTT<br>CCGGACATGCACTTTAA	
F3	TTAAAGTGCATGTCCGGAAGTG<br>ATACACAAGGCTTTGA	
F4	TGCGCCGTTGATTCAAAAG<br>TGACATTAGAAAACCGA	
F5	TCGGTTTTCTAATGTCACTT<br>TTGAATCAACGGCGCA	
F6	AGCATTTCGGACTTTCTGTCG	
J1	TTCTTATAAAGAGCGGTCG	95 °C for 5 min，30 cycles of 95 °C for 60 s, 55-63 °C for 30 s, 72 °C for 60 s; 72 °C for 10 min
J2	GCGGTCCATATATACTCCGTAAATT<br>TTCCGGCATAGATATCGAAAAA	
J3	TTTTTCGATATCTATGCCGGAAAATT<br>TACGGAGTATATATGGACCGC	
J4	TCAAAGCCTTGTGTATCATTATAA<br>CAGCTCTTCATACGTTTTCATCTC	
J5	GAGATGAAAACGTATGAAGAGCTGT<br>TATAATGATACACAAGGCTTTGA	
J6	CATTTGCGCGTTTGATTCACG<br>TGACATTAGAAAACCGA	
J7	TCGGTTTTCTAATGTCACGTGA<br>ATCAAACGCGCAAATG	
J8	GTGCGGAACGGCATGGAATTAA	
JD1	TGTACGCCTACGATCTGTCTTC	95 °C for 5 min, 30 cycles of 95 °C for 1 min, 60°C for 1 min, 72°C for 2 min; 72°C for 10 min
JD2	CTTTAACCTGCCTGTTCACGA	
JD3	CCCGCCTCAAGAGTGATTCAT	95 °C for 5 min，30 cycles of 95 °C for 1 min, 57 °C for 30 s, 72 °C for 1 min; 72 °C for 10 min
JD4	ACAGCCGCAGTGAAAGCAT	

Table S4 Theoretical molecular mass of lipopeptides and corresponding m/z value

Lipopeptides	M	$[M+H]^+$	$[M+Na]^+$	$[M+K]^+$
C_{13}IturinA	1028.5274	1209.5352	1051.5172	1067.4911
C_{14}IturinA	1042.5430	1043.5508	1065.5328	1081.5067
C_{15}IturinA	1056.5586	1057.5664	1079.5484	1059.5223
C_{16}IturinA	1070.5742	1071.5820	1093.5640	1109.5379
C_{17}IturinA	1084.6051	1085.5976	1107.5796	1123.5535
C_{18}IturinA	1098.6054	1099.6132	1121.5952	1137.5691
C_{13}IturinB	1129.5114	1030.5192	1052.5012	1068.4751
C_{14}IturinB	1043.5270	1044.5348	1066.5168	1082.4907
C_{15}IturinB	1057.5426	1058.5504	1080.5324	1096.5063
C_{16}IturinB	1071.5582	1072.5660	1094.5480	1110.5219
C_{17}IturinB	1085.5738	1086.5816	1108.5636	1124.5375
C_{18}IturinB	1099.5894	1100.5972	1122.5792	1138.5531
C_{14}Mycosubtilin	1042.5430	1043.5508	1056.5328	1081.5067
C_{15}Mycosubtilin	1056.5586	1057.5664	1079.5484	1095.5223
C_{16}Mycosubtilin	1070.5742	1071.5820	1093.5640	1109.5379
C_{17}Mycosubtilin	1084.5898	1085.5976	1107.5796	1123.5535
C_{14}BacillomycinD	1030.5317	1031.5359	1053.5215	1069.4954
C_{15}BacillomycinD	1044.5473	1045.5551	1067.5371	1083.5110
C_{16}BacillomycinD	1058.5629	1059.5707	1081.5527	1097.5266
C_{17}BacillomycinD	1072.5785	1073.5863	1095.5683	1111.5422
C_{14}BacillomycinF	1056.5586	1057.5664	1079.5484	1095.5223
C_{15}BacillomycinF	1070.5742	1071.5820	1093.5640	1109.5379
C_{16}BacillomycinF	1084.5898	1085.5976	1107.5796	1123.5535
C_{17}BacillomycinF	1098.6054	1099.6132	1121.5952	1137.5691
C_{14}BacillomycinL	1020.5110	1021.5188	1043.5008	1059.4747
C_{15}BacillomycinL	1034.5266	1035.5344	1057.5164	1073.4903
C_{16}BacillomycinL	1048.5422	1049.5500	1071.5320	1087.5059
C_{17}BacillomycinL	1062.5578	1063.5656	1085.5476	1101.6133
C_{13}SurfactinA	1007.6496	1008.6574	1030.6394	1046.6133
C_{14}SurfactinA	1021.6652	1022.6730	1044.6550	1060.6289
C_{15}SurfactinA	1035.6808	1036.6886	1058.6706	1074.6445
C_{16}SurfactinA	1049.6964	1050.7042	1072.6862	1088.6601
C_{13}SurfactinB	999.6340	999.6418	1016.6238	1032.5977
C_{14}SurfactinB	1007.6496	1008.6574	1030.6394	1046.6133

C_{15}SurfactinB	1021.6652	1022.6730	1044.6550	1060.6289
C_{16}SurfactinB	1035.6808	1036.6886	1058.6706	1074.6445
C_{13}SurfactinC	1007.6496	1008.6574	1030.6394	1046.6133
C_{14}SurfactinC	1021.6652	1022.6730	1044.6550	1060.6289
C_{15}SurfactinC	1035.6808	1036.6886	1058.6706	1074.6445
C_{16}SurfactinC	1049.6964	1050.7042	1072.6862	1088.6601
C_{14}FengycinA	1434.7620	1435.9698	1457.7518	1473.7257
C_{15}FengycinA	1448.7776	1449.7854	1471.7674	1487.7413
C_{16}FengycinA	1462.7932	1463.8010	1485.7830	1501.7569
C_{17}FengycinA	1476.8088	1477.8166	1499.7986	1515.7725
C_{18}FengycinA	1490.8244	1491.8322	1513.8142	1529.7881
C_{14}FengycinB	1462.7932	1463.8010	1485.7830	1501.7569
C_{15}FengycinB	1476.8088	1477.8166	1499.7986	1515.7725
C_{16}FengycinB	1490.8244	1491.8322	1513.8142	1529.7881
C_{17}FengycinB	1504.8400	1505.8478	1527.8298	1543.8037
C_{18}FengycinB	1518.8556	1519.8634	1541.8454	1557.8193
